# Impact of *in vitro* fertilization by refrigerated versus frozen buffalo semen on developmental competence of buffalo embryos

**DOI:** 10.1590/1984-3143-AR2020-0033

**Published:** 2020-11-24

**Authors:** Jaci Almeida, Beatriz Parzewski Neves, Mayara Ferreira Brito, Robson Ferreira Freitas, Lílian Gabriel Lacerda, Lira Santos Grapiuna, João Paulo Haddad, Patrícia Alencar Auler, Marc Henry

**Affiliations:** 1 Departamento de Clínica e Cirurgia Veterinária, Escola de Veterinária, Universidade Federal de Minas Gerais, Campus Pampulha, Belo Horizonte, MG, Brasil; 2 Laboratório BH Embriões, Belo Horizonte, MG, Brasil; 3 Departamento de Medicina Veterinária Preventiva, Escola de Veterinária, Universidade Federal de Minas Gerais, Campus Pampulha, Belo Horizonte, MG, Brasil

**Keywords:** oocyte donors, OPU, *in vitro* fertilization

## Abstract

The objective of this study was to evaluate the fertility of buffalo semen for *in vitro* embryo production (IVEP) by comparing the effectiveness of refrigerated versus frozen semen. Three OPU sessions were held at 30-day intervals. For oocyte fertilization three buffalo bulls were used, one per session. At each OPU-IVEP session, one ejaculate was collected and divided into two equal aliquots. Each aliquot was either refrigerated at 5ºC/24 hours or frozen. A TRIS extender containing 10% low density lipoproteins, 0.5% lecithin and 10 mM acetylcysteine was used adding 7% glycerol for freezing. Sperm motility/kinetic was evaluated by CASA and sperm membrane integrity by the hypoosmotic swelling test. The evaluations were performed at 0 h (post final dilution at 37ºC), at 4 and 24 hs post-incubation at 5ºC and post-thaw. At 24 hs incubation and immediately post thaw sperm cells were used for *in vitro* fertilization of buffalo oocytes equally distributed between both groups. Cleavage rates and embryo development were followed. The embryo/matured and embryo/cultured rates were 25.4 x 14.0% and 29.4 x 18.5% (P<0.05), for chilled and frozen semen, respectively. It is concluded that cooled semen can be used for *in vitro* embryo production in buffalo and that a better efficiency may be expected for cooled compared to frozen semen.

## Introduction

Since the first pregnancy in buffalo resulting from an embryo produced *in vitro* (Madan et al., 1992) and the first calf born in Brazil (Ohashi et al., 2006) reproductive biotechnologies in buffalos such as intracytoplasmic sperm injection (ICSI), transgenes, cloning and even ovum pick-up (OPU) in two months old buffalo calf have been done (Bernardes, 2017). All this resulted in some parts of the world in commercial exploitation of *in vitro* production of embryos (IVEP) in Buffalos (ABS Brasil, 2016). From 1991 to 2008 more than 100 publication have been released reporting high oocyte maturation index (80%), moderate cleavage rate (50%) and moderate to low blastocyst rate (20%) in the buffalo specie (Surresh et al., 2009 and Soliman et al., 2018). However, the use of the technique in this species still faces some limitations. The production of embryo rates per egg collection section is still low (Salzano et al., 2018; Baruselli et al., 2020), thus increasing the cost of pregnancy 3 to 4 times when compared to what was achieved in zebu (Ohashi et al., 2017).

Efficiency (OPU/IVEP) in buffalo is necessary to enhance the use of superior genetic material. OPU can be performed on prepubertal, anestrus, cycling and even pregnant buffalo, however, some anatomophysiological peculiarities, such as lower weight and size of ovaries compared to cows (Vitoria, 1997), lower total primordial follicular number in buffalo when compared to cows (Danell, 1987; Erickson, 1966; Baruselli et al., 1997; Gimenes et al., 2009; Campanile et al., 2010; Santos et al., 2013 and Liang et al., 2016), greater follicular rate atresia in buffaloes (Le Van Ty et al., 1989; Ocampo et al., 1994; Perera, 2011; Santos et al., 2013), low eligibility rate for buffalo oocytes (38.4%) for the culture due to less compaction and fewer cumulus layers (Ohashi et al., 2003; Gimenes et al., 2010; Marin et al., 2019) and the seasonality effects on the quality of oocytes (Gimenes et al., 2010; Gasparrini, 2018) are limiting factors for *in vitro* embryo production (IVEP) in buffalo species (Salzano et al., 2018; Baruselli et al., 2020).

Among all attempts to improve the efficiency of the IVEP no reports were found proposing the use of oocyte fertilization by cooled semen. The process of freezing semen is known to “pre-capacitate” sperm cells which is an advantage for the IVEP. Cooling sperm cells, at least in the beginning of the incubation period, result in less injury to the sperm cells which could not induce the pre-capacitate stage required for IVF. To our knowledge, there are no reports in buffalo using cooled and comparing cooled to frozen semen for *in vitro* fertilization. As each steps of the process of IVEP may contribute for general improvement the aim of the present study was to evaluate the efficiency of the use of cooled semen in substitution of frozen semen to produce buffalo embryo *in vitro*.

## Material and methods

### Ethics

This study was approved by the Ethics Committee for Domestic Animals of the Universidade Federal de Minas Gerais - UFMG (protocol CEUA UFMG 368/2015), following the ethical principles for experiments with animals.

### Site of the experiment and execution period

The experiment was carried out at the “Centro de Biotecnologia em Bubalinocultura”, Fazenda Modelo – Pedro Leopoldo – Estado de Minas Gerais, Brasil at a 710 m altitude, 19^o^37'05” South latitude and 44o02'55 West longitude. Ovum pick-up sessions were performed from September to November at 30 days intervals.

### Animals

Three Murrah bulls (*Bubalus bubalis*) of average five years old, body condition score = 4 (1-5) and average weight of 920 kg were used as semen donors. All were maintained isolated from females and had good fertility records.

OPU sessions were performed in 25 crossbred buffalo (Murrah x Mediterranean) aging 3 to 11 years, body condition score 3 to 5 (1-5) and average body weight of 625 kg. Two third were lactating females and all were approved at the gynecological evaluation. The herd was maintained at pasture with mineral supplementation and water *ad libitum*.

### Semen collection and evaluation

Three seminal donors were conditioned to artificial vagina using a buffalo cow as dummy and were under a weekly collection schedule for more than a year period before the beginning of the experiment. Each seminal collection attempt was preceded by a fake mount, aiming higher sperm concentration. For each OPU session (n=3) a different seminal donor was used.

*Immediate evaluations*: motility was evaluated subjectively under a light microscope (200x) and samples for concentration and sperm morphology were taken. Sperm samples for concentration were fixed in buffered formol-saline solution (Hancock, 1957) in a 1:200 dilution and count was performed using a hemocytometer chamber (Neubauer) under light microscope (400x) (CBRA, 2013). Sperm morphology was evaluated (200 cells) under phase contrast microscope (1000x). Minimal quality requirements to use ejaculates were total motility ≥ 70%; sperm morphology ≥80 normal; sperm concentration ≥ 0.9x10^9^ spermatozoa/mL (CBRA, 2013).

*Post seminal handling evaluations*: sperm motility was evaluated by computer assisted analyzer (Sperm Class Analyzer - SCA^®^ v.4.0) immediately post- final dilution (0 h), after 4 and 24 h. of incubation at 5^o^C and post-thaw (frozen aliquots) after 5 minutes of incubation at 37°C. Setups of the SCA were: particle area 20 a 70 microns^2^, VCL – 10 < Slow <25; > 25 Medium < 50; Progressivity - > 70% straightness, Circular - < 50% LIN, VAP – average of 5 readings and connectivity - 12. Previous to CASA evaluation an alliquot of each sample was incubated at 37^o^C for 5 min and thereafter a 5 µL volume was placed in between a prewarmed (37^o^C) slide and coverslide. Final results expressed the average of 5 fields with a minimum of 200 cells per field. Total motility (TM %), progressive motility (PM %), curvilinear velocity (VCL µm/sec.), average path velocity (VAP µm/sec.), straight line velocity (VSL µm/sec.), linearity (LIN %), straightness (STR %), wobble (WOB %), lateral head displacement (ALH µm) and beat cross frequency (BCF hz) were evaluated. All those parameters were found best correlated with fertility by Farrell et al. (1998). Functional integrity of the plasma membrane was evaluated by the hypoosmotic swelling test (HOST). Fifty microliters of each sample were added to 500 μL of a hypoosmotic fructose-sodium citrate solution (121 mOsm/L – 2.702g of fructose + 1,324g of sodium citrate + distillated water q.s. 100 mL) and incubated for 30 min. at 37 ºC. Thereafter samples were fixed adding 200 μL of buffered formol saline. Two hundred cells were examined using a phase contrast microscope (1000x) (Loaiza-Echeverri et al., 2015).

### Semen handling

Post collection, seminal samples were maintained at 37°C until final dilution. Each ejaculate was divided in two aliquots, both diluted to obtain 50x10^6^ sperm/mL using TRIS extender with no glycerol for cooling and with 7% glycerol for freezing. Diluted seminal samples were packed in 0.5 mL straws (IMV^®^ Techgias, L'Aigle Cedex, França), and cooling was initiated submerging straws protected in a plastic bag into a recipient with 1.4 L of water at 27°C. A cooling rate of -0.25^o^C/min. down to 5^o^C was obtained submerging the water recipient (27°C) in a basin with 10 L o water at 5^o^C. Samples aimed at cooling were maintained at 5^o^C until the fertilization process (~24hs) while samples aimed at freezing were maintained at 5^o^C for 4 hours before freezing. Straws were then frozen in a Styrofoam box (39x19x30 cm), 5 cm above the nitrogen (N_2_) level for 20 minutes, thereafter they were plunged into the N_2_. Minimal requirements to use the semen was ≥ 50% motility for cooled semen and ≥ 30% of motility for frozen semen.

### Seminal extender production

Extender contained TRIS (2.42 g), citric acid (1.36 g), fructose (1.0 g), low density lipoproteins (LDL -10 mL), acetylcysteine 10 mM, 0.5% of soya lecithin (Solec FP-40^®^ - Solae), Equex 0.4%, amikacin (83.4µg/mL), and autoclaved Milli-Q^®^ H_2_O (s.q.f. 100 mL). All reagents were Sigma-Aldrich^®^. Half of each batch was used for cooling and the other half was used for freezing semen adding 7% of glycerol. Extender was preserved cooled at 5^o^C for immediate use (within 24 h.) and was kept frozen (-20^o^C) until just prior subsequent uses. Low density lipoproteins were extracted from fresh chicken eggs following the technique proposed by Moussa et al. (2002) with few modifications suggested by Neves (2008).

### *In vitro* production of embryos

*OPU and initial handling*: a total of three OPU sessions were done by an experienced technician. At each session 16 to 25 female buffalo were used and average interval between sessions was 30 days. For OPU donors were immobilized in a cattle crush and 2 mL of lidocaine 2% (Lidovet^®^, Bravet) was used for low epidural anesthesia. A Mindray^®^ DP-2200 ultrasound linked to a micro convex multifrequency probe adjusted to 7.5 MHz frequency was used for OPU. Aspiration guide (WTA - Watanabe Tecnologia Aplicada^®^) was coupled to the probe and a 18G needle (WTA - Watanabe Tecnologia Aplicada^®^) linked to a Teflon duct of 1.8 mm diameter and 1.2 m long were used to collect the follicular fluid.

Before each sessions all ducts were washed with prewarmed (37°C) Dulbeco modified phosphate buffered saline (DMPBS - Biodux^®^), added with 1 mL of suine sodium heparin (5.000 UI/mL; Hepamax-s^®^, BLAU Pharmaceutics S.A.) per liter of DMPBS. Eigthy five mmHg vacuum pressure (12 to 15 mL of water/minutes) was used for follicular aspiration. All follicles of ≥ 2,0 mm diameter were aspirated, and follicular fluids were gathered in conic prewarmed flasks. Under a controlled ambiance, immediately after aspiration of a group of 4 donors, follicular fluid was filtered (embryo filter - WTA - Watanabe Tecnologia Aplicada^®^) and oocytes were then washed in 70 µL drops of TCM 199 Hepes + 10% bovine fetal serum (BFS) + 22µg/mL pyruvate + 83,4 µg/mL amikacin and thereafter conditioned in 2 mL cryotubes containing the same media. Cryotubes were immediately placed and subsequently transported to the laboratory in an incubator (WTA - Watanabe Tecnologia Aplicada^®^) at 38°C. Maximal time elapsed between first ovum pickup and arrival at the laboratory was 5 hs.

*Oocyte laboratory handling*: oocytes were matured in TCM-199 supplemented with BSA, antioxidants, hormones, amikacin and BFS and incubated at 38.8°C with 5% CO_2_ atmosphere for 22 hours. Thereafter oocytes were washed in Tyrode´s-albumin-lactate-pyruvate (TALP) media and divided in two groups equally balanced according to their quality parameters as described by Di Francesco et al. (2011) for buffalos. For the fertilization process, either with cooled or frozen semen, oocytes were accommodated in a 70 µL TALP + BSA drop supplemented with heparin, PHE solution and amikacin under mineral oil and incubated at 38.8°C with 5% CO_2_ for 21 hours.

**Figure 1:**
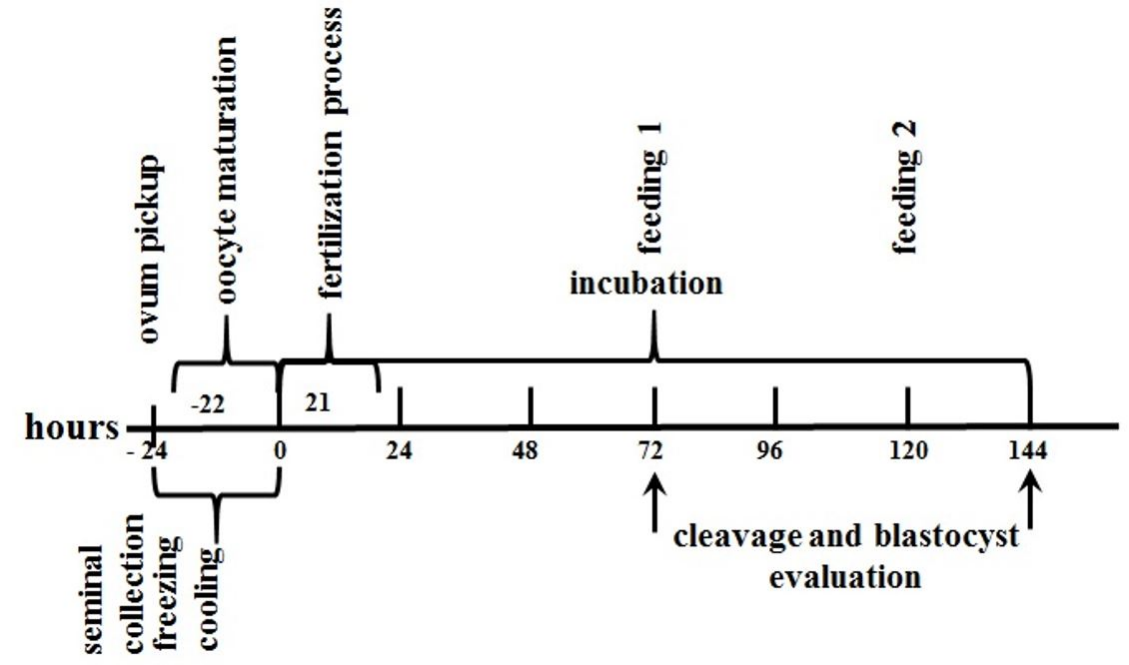
Timeline of PIVE stages in buffalo.

Sperm cells were selected by two consecutive centrifugations. Firstly, semen was gently dropped over a percoll solution previously warmed to 38.8°C for 5 minutes and centrifuged for two minutes at 5500 RPM. The supernatant was withdrawn and one mL of TALP was added for the second centrifugation as previously described. Eight µL of the precipitate was added in each drop containing oocytes. After the fertilization incubation period presumable zygotes of both groups were denuded and incubated under mineral oil in 60 µL of synthetic oviductal fluid (SOF) supplemented with BSA, amino acids, antioxidants, amikacin and BFS in a 5% CO_2_, 5% O_2_, 90% N and saturated humidity atmosphere at 38,8°C. Feedings were performed on days three and five withdrawing 45 µL of the culture media and adding the same amount of SOF. Cleavage and blastocyst rates were evaluated, respectively, on days three and six (Figure 1).

### Statistical analysis

Seminal data were analyzed by the Statacorp software (Statacorp, 2012) and the T test was used for independent samples. For IVEP data, a descriptive analysis was included, and the “Z” test was used.

## Results

Characteristics of buffalo’s ejaculates evaluated immediately post collections are presented in table 1 and kinetics parameters of diluted (immediately post final dilution), cooled and post-thaw sperm cells used for *in vitro* production of embryos and evaluated by the Sperm Class Analyzer - (SCA^®^ v.4.0) are shown in Table 2.

**Table 1:** Buffalo’s ejaculates characteristics evaluated immediately post-collection

Buffalo	Volume (mL)	Mass motility (0-5)	Motility (%)	Vigor (0-5)	Concentration (x10^6^/mL)	MD (%)	N (%)
1	1.5	3	80	4	1350	9	87
2	1.8	3	90	4	1580	10	83
3	2.1	3	80	3	930	8	88,5
**Mean ±SD**	**1.8±0,3**	**3.0±0,0**	**83.3±5,8**	**3.7±0.6**	**1286.7±329.6**	**9.0±1.0**	**86.1±2.8**

MD = major defects; N= morphologically normal sperm cells and SD = Standard deviation.

**Table 2:** **-** Mean and standard deviation of kinetics parameters of buffalo’s spermatozoa evaluted by the CASA at time “0” (immediately post-dilution before cooling), 4 and 24 hs after cooling (5^o^C) and immediately post thaw.

Kinetics parameters	Time of observation			
	0h	4h	24h	PT
TM (%)	97.0±1.0^a^	95.0±1.0^a^	90.3±2.1^b^	60.3±3.2^c^
PM (%)	85.3±915^a^	77.3±6.1^b^	64.3±6.0^c^	41.3±4.0^d^
VCL (µm/s)	104.8±15.9^a^	94.3±0.5^b^	88.4±7.6^b^	53.9±0.7^c^
VAP (µm/s)	72.8±7.3^a^	66.0±5.4^b^	63.8±5.1^b^	42.1±0.8^c^
VSL (µm/s)	49.2±5.6^a^	46.2±10.3^a^	44.4±9.7^b^	32.3±1.2^c^
LIN (%)	47.9±10.1^b^	49.5±11.6^b^	49.3±7.2^b^	59.3±1.1^a^
STR (%)	67.9±8.6^b^	69.0±10.0^b^	69.0±11.2^b^	79.4±0.6^a^
WOB (%)	70.0±6.7^b^	70.1±6.1^b^	71.8±5.1^b^	77.3±1.5^a^
ALH (µm)	3.7±0.6^b^	3.3±0.2^b^	3.2±0.2^b^	2.2±0.1^a^
BCF (Hz)	9.3±1.0^b^	10.1±0.6^a^	10.4±1.8^a^	10.0±0.2^a^

Average of 3 donors, 1 ejaculate per donor; samples were pre warmed to 37°C; different letters in the row indicates p<0,05; PT: post-thaw; TM: total motility; PM: progressive motility; VCL: curvilinear velocity; VAP: average path velocity; VSL: straight line velocity; LIN: Linearity; STR: straightness; WOB: wobble; ALH: lateral head displacement; BCF: beat cross frequency.

Sperm plasmatic membrane integrity evaluated by the HOST (% reactive cells) was 94.1±2.9^a^, 92.2±3.2^a^, 89.9±1.5^a^ and 58.6±2.5^b^% (mean±standard deviation) respectively for immediately post-dilution (37ºC), after 4 and 24 hours at 5^o^C and immediately post thaw . Values did not decrease after 4 or 24 hours of cooling but was less for frozen-thawed sperm cells (P<0.05).

The OPU session (n=3) yield and the efficiency of IVEP in buffalos per OPU session and type of semen (cooled x frozen) are presented in Table 3. It can be observed that despite OPU sessions were performed out of the local physiological breeding period, in all three attempts follicular growth activity on the ovaries allowed a rather good success of oocytes recovery. Additionally, IVEP efficiency was higher when cooled semen was used in comparison to frozen semen.

**Table 3:** Ovum pick up (OPU) in bufalos (*Bubalus bubalis*) - results per session (n=3 - thirty days inter-sessions interval - from September to November) and *in vitro* embryo production (IVEP) using cooled or frozen sperm cells.

OPU variables			OPU Session						
			1ª		2ª			3ª	
Nº of aspirated donors			20		25			16	
Nº of aspirated follicles			161		140			139	
Average number of follicles/donor			8.1		5..6			8.7	
Total number of retrieved oocytes			130		105			94	
Oocyte recovery rate (%)			81		75			68	
Nº of retrieved viable oocytes (grading I, II e III)			102		80			57	
No viable oocyte rate (%)			28		25			27	
Viable oocyte rate (%)			78.5		76.1			47.4	
Average retrieved oocyte number/donor			6.5		4.2			5.9	
**OPU variables**	**OPU Session**						**Total**		
	**1ª**		**2ª**		**3ª**				
	**Semen type**		**Semen type**		**Semen type**				
	**CS**	**FS**	**CS**	**FS**	**CS**	**FS**	**CS**		**FS**
Nº of matured oocytes	51	51	40	40	27	30	118		121
Nº of cultured oocytes	41	30	34	32	27	30	102		92
Nº of cleaved oocytes	16	13	11	6	8	4	35^a^		23^b^
Nº of produced embryos	16	12	7	5	7	0	30^a^		17^b^
% of cleaved/cultured oocytes	31	24	18	13	18	0	25.4^a^		14.0^b^
% of embryos/matured oocytes	39	40	21	16	26	0	29.4^a^		18.5^b^
% of embryos/cleaved oocytes	100	92	64	83	88	0	85.7		73.9

Different letters in a row indicates P<0.05; CS: cooled semen; FS: frozen semen; comparison was made using the Z test. Total = Session 1+2+3

## Discussion

The use of cooled semen for *in vitro* production of buffalo embryos showed to be an effective alternative compared to the use of frozen semen.

As far as seminal characteristics of the three seminal donors is concerned values were within the expected range for the species (CBRA, 2013; Henry et al.*,* 2017). Besides that, seminal parameters *per se* expressed good fertilization potential (Severo, 2009), it is worth to stress that donors were under a weekly long-lasting seminal collection schedule which lessen aging sperm cells within the epididymis tail favoring good sperm quality.

The aim of choosing 24 hours of cooling was based on the speculation that this length of time would better represent what is feasible to transport semen from a farm (origin of a seminal donor) to a IVEP laboratory, this, considering the extension of the country were the experiment was achieved. As expected, a decrease in TM, PM, VCL, VAP and in VSL was observed in post thaw sperm cells and in cells maintained at 5°C for 24 hs. The drop was more substantial for frozen/thawed sperm cells (fluctuating between 35 and 50% - (P<0.05) while the major drop for cooled sperm cells was about 25% for PM and velocity parameters decreased by 15% or even less among seminal donors. In both type of semen, VCL, VSL, LIN and ALH values did not characterize any sperm hyperactivation (Gillan et al., 2008) which could be detrimental for the whole process.

Sperm characteristics after thaw were within the range reported for buffalos by (El-Sisy et al., 2007; Mahmoud et al., 2013, 2015b) and after 24hs of incubation at 5°C similar to the studies of Akhter et al. (2011); Singh et al. (2012); Almeida et al. (2016); Becerra (2017). Additionally, sperm kinetics parameters related to fertility described by Matos et al. (2008) (VCL and ALH), Mortimer (2000) and Verstegen et al. (2002) (VCL, VAP, VSL, BCF and LIN) were within an acceptable range before the fertilization procedure. Thus, the fertilization capacity of sperm cells preserved either by cooling or freezing was promising, particularly considering that *in vitro* fertilization was the goal.

OPU sessions in the present study occurred from September to November. Average follicle punctured per donor varied from 5.6 to 8.7 which is close to numbers found by Sá et al. (2009), Gasparrini et al. (2014) and Pereira (2015). A higher number of follicles per donor (from 12.6 to 15.6) was reported by Ferraz et al. (2015), Ohashi et al. (2017) and Marin et al. (2019) selecting donors with higher number of follicles, reported an average 13.6 follicle per female. Average number of recovered oocytes per donor and session varied from 4.2 to 6.5 above the number (n=3.7) found by Pereira (2015), while Ohashi et al. (2017) and Marin et al. (2019), using a population of donors with higher number of follicles found an average of 7.8 and 10.2 oocytes per donor, respectively. Oocyte recovery rate obtained in the present study (68 to 81%) was above that reported by Sá et al. (2009) (57.7%) and Pereira (2015) (35%).

Considering the efficiency of the OPU obtained by the end of each session, including oocyte yield and quality oocytes grading, in all three attempts it was decided to proceed with the IVEP. Thus, the oocytes collected were considered adequate to analyze comparatively the efficiency of using cooled versus frozen sperm cells.

Values obtained to percentage of cleaved/cultured oocytes, percentage of embryos/matured oocytes, percentage of embryos/cultivated oocytes and of embryos/cleaved oocytes are within the expected values for the species. Cleaved rate of 41.7%; 67.5%; 38.7% (October to February), 63.4% (March to June) and 33.4% were reported by Gasparrini et al. (2014), Pereira (2015) and Ferraz et al. (2015), respectively. This indicates that our laboratory procedures were consistent with the ongoing procedures.

### Cooled sperm and fertilization capacity

The feasibility of the use of cooled semen to fertilize buffalo oocytes *in vitro* was evident based on percentage of cleaved/cultured oocytes (34.3%) and of embryos/cleaved oocytes (85.7%). These results were obtained even using the three types of oocytes quality grading (I to III) which maintained a similar proportion for the three OPU session. Additionally, no special laboratory process was used to prepare cooled sperm cells for the fertilization capacity test. Efficiency was similar for the three seminal donors despite that seminal different fertilizing capacity *in vitro* may be expected between buffalo seminal donors (Totey et al., 1992; Zicarelli et al., 2009). In natural breeding or conventional artificial insemination difference in fertility between bulls has been reported (Saacke et al., 1998). Thus, the process of cooling instead of freezing did not alter/suppress sperm cells ability to capacitate and go through acrosome reaction and fertilize the oocytes.

### Fertilization capacity of cooled x frozen/thawed sperm cells

The *in vitro* fertilization capacity of cooled semen was superior to that of frozen/thawed semen. This was observed by the superior index of embryos/matured (25.4x14.0%) and of embryos/cultivated oocytes (29.4x18.5%) in favor of the cooled sperm cells (P<0.05). Once oocytes were fertilized the percentage of embryos per cleaved oocyte was similar between type of sperm cells. An effect of ejaculate can be excluded once the same ejaculate of each seminal donor was used simultaneously to either cool or freeze. Additionally, the same trend was observed for the three seminal donors, i.e. cooled semen was more efficient than frozen semen, this altogether minimize the possibility of an effect of seminal donor. Number of motile sperm cells available for the fertilization as major effect on the results should be minimized or even withdraw considering that through the percoll method a similar number of sperm cells were selected to be incubated with oocytes.

As sperm cells preserved by the two methods were previously selected through the percoll method for the process of fertilization it could be expected that differences found in kinetics parameter between the two types of semen would be minimized or even expunged. It is known that sperm cells submitted to the freezing/thawing processes are more likely to suffer cryoinjuries during this process and has a shorter *in vitro* lifetime that when only cooling is used (Watson, 1995; Bucher et al., 2009; Mahmoud et al., 2015a), particularly for a relatively short period as 24 hours. The motility of cooled semen (5°C) in several studies could be maintained above 30% for up to 3 days Singh et al. (2012), Almeida et al. (2016), Becerra (2017) and Almeida (2018), indicating that 24 hs, of incubation was not a crucial length of time as to the maintenance of sperm activity.

In the present study the incubation of oocytes with sperm cells for the occurrence of fertilization lasted for 21 hours. Even though no specific control along the process of incubation was done, it is reckless to attribute the lower efficiency of the frozen semen exclusively to the life span of the cells; differences in sperm kinetics could account for this, however, as cited above, selection of sperm cells by the percoll method was done beforehand and have the theoretical potential to minimize this effect. Therefore, cryoinjuries caused by the freezing/thawing processes other than those already mentioned cannot be excluded as causing factors, alone or in association.

## Conclusion

It is concluded that the semen chilled for 24 hours at 5ºC proved to be effective in the fertilization of buffalo oocytes by the PIVE technique, presenting results superior to the use of frozen semen.
